# Quantifying cardiac‐induced brain tissue expansion using DENSE

**DOI:** 10.1002/nbm.4050

**Published:** 2018-12-21

**Authors:** Ayodeji L. Adams, Hugo J. Kuijf, Max A. Viergever, Peter R. Luijten, Jaco J.M. Zwanenburg

**Affiliations:** ^1^ Department of Radiology University Medical Center Utrecht Utrecht The Netherlands; ^2^ Image Sciences Institute University Medical Center Utrecht Utrecht The Netherlands

**Keywords:** brain tissue motion, DENSE, pulsatility, small vessel disease

## Abstract

Brain tissue undergoes viscoelastic deformation and volumetric strain as it expands over the cardiac cycle due to blood volume changes within the underlying microvasculature. Volumetric strain measurements may therefore provide insights into small vessel function and tissue viscoelastic properties. Displacement encoding via stimulated echoes (DENSE) is an MRI technique that can quantify the submillimetre displacements associated with brain tissue motion. Despite previous studies reporting brain tissue displacements using DENSE and other MRI techniques, a complete picture of brain tissue volumetric strain over the cardiac cycle has not yet been obtained. To address this need we implemented 3D cine‐DENSE at 7 T and 3 T to investigate the feasibility of measuring cardiac‐induced volumetric strain as a marker for small vessel blood volume changes. Volumetric strain over the entire cardiac cycle was computed for the whole brain and for grey and white matter tissue separately in six healthy human subjects. Signal‐to‐noise ratio (SNR) measurements were used to determine the voxel‐wise volumetric strain noise. Mean peak whole brain volumetric strain at 7 T (mean ± SD) was (4.5 ± 1.0) × 10^−4^ (corresponding to a volume expansion of 0.48 ± 0.1 mL), which is in agreement with literature values for cerebrospinal fluid that is displaced into the spinal canal to maintain a stable intracranial pressure. The peak volumetric strain ratio of grey to white matter was 4.4 ± 2.8, reflecting blood volume and tissue stiffness differences between these tissue types. The mean peak volumetric strains of grey and white matter tissue were found to be significantly different (*p* < 0.001). The mean SNR at 7 T and 3 T of the DENSE measurements was 22.0 ± 7.3 and 7.0 ± 2.8 respectively, which currently limits a voxel‐wise strain analysis at both field strengths. We demonstrate that tissue specific quantification of volumetric strain is feasible with DENSE. This metric holds potential for studying blood volume pulsations in the ageing brain in healthy and diseased states.

**Abbreviations used**
AP: anterior to posterior
CoV: coefficient of variation
CSF: cerebrospinal fluid
cSVD: cerebral small vessel disease
*D*_enc_: displacement encoding
DENSE: displacement encoding via stimulated echoes
ERB: ethical review board
FH: feet to head
IQR: inter‐quartile range
POx: pulse oximeter
RL: right to left
ROI: region of interest
SENSE: sensitivity encoding
SNR: signal‐to‐noise ratio
VCG: vectorcardiogram
WET: water suppression enhanced through *T*_1_ effects

## 1 INTRODUCTION

The brain is mechanically coupled to the heart, which results in pulsatile viscoelastic deformation and volumetric strain of the brain tissue over the cardiac cycle. In a healthy brain, the small vessels embedded within the brain tissue temporarily swell to accommodate the increased intracranial blood volume during systole, thereby causing brain expansion. These cardiac‐induced changes in brain tissue volume (i.e. volumetric strain) should therefore reflect transient changes in the blood volume of the small vessels (small arteries/veins, arterioles/venules and capillaries), as well as the material properties of the tissue surrounding the small vessels. Non‐invasive assessments of brain tissue motion may thus provide valuable information on small vessel function or allow the derivation of the viscoelastic properties of brain tissue.^1^ Brain tissue and vessel stiffness naturally change with age,^2^, ^3^ which suggests that brain tissue motion particularly holds potential as a means to study the small vessels and/or the viscoelastic properties of the brain as a function of age. Conceivably, it may permit the study of the brain in the transition from healthy to diseased states, such as that which occurs in the development of cerebral small vessel disease (cSVD), where damage to the small vessels causes further damage to the tissue. Unlike the larger arteries of the brain or the macroscopic tissue damage that arises from cSVD, the small vessels themselves are difficult to study in vivo with current neuroimaging techniques. Brain tissue motion may provide a valuable biomarker for the disease with the potential to provide an indirect window to the small vessels that are involved in cSVD, and to the microscopic tissue changes subsequent to cSVD as reflected in tissue stiffness.

The overall phenomenon of brain tissue motion is subtle, involving submillimetre displacements^4^ and very small flow changes in the microvasculature.^5^ General improvements in available hardware, software and imaging techniques since the early exploration of brain tissue motion using MRI^6^, ^7^, ^8^, ^9^ have allowed renewed interest in the phenomenon of brain tissue motion in healthy subjects as well as patients.^10^, ^11^ Displacement encoding via stimulated echoes (DENSE), a non‐invasive MRI technique, was shown to be a feasible method for capturing the displacement vector fields that characterize brain tissue pulsatility,^4^, ^12^, ^13^ thereby allowing the derivation of brain tissue volumetric strain. However, despite the important potential of brain tissue volumetric strain in evaluating small vessel function, a complete picture of its variation over the cardiac cycle has not yet been obtained. Previous investigations of brain tissue motion did not assess brain tissue volumetric strain,^4^ or reported it only in relatively small regions of interest (ROIs) within at most a few slices,^14^, ^15^ or measured only 2D strain.^13^ These measurements (even those made at 3 T) also probably suffered considerably from noise, since utilizing DENSE to encode brain tissue motion results in an inherent 50% signal loss due to the use of a single stimulated echo in the acquisition window.^16^ Additionally, the derivation of volumetric strain requires the computation of spatial derivatives, which amplifies the noise present in the displacement maps. Therefore, the relationship between the signal‐to‐noise ratio (SNR) of DENSE measurements and the calculated brain tissue volumetric strain needs to be assessed in order to identify the uncertainty in the computed volumetric strain maps.

In this study, we implemented high‐resolution 3D cine‐DENSE at 7 T, and also at 3 T for reference. The goal was threefold: first, to obtain (in healthy subjects) whole brain volumetric strain measurements covering the entire cardiac cycle, which we propose as an important metric reflecting small vessel blood volume pulsations; second, to demonstrate the potential of DENSE in practice by assessing the physiological pulsatile differences between grey and white matter tissue (the volumetric strain values obtained in this study were evaluated against literature values of cerebrospinal fluid (CSF) stroke volumes and against literature values of the relative blood volume difference between grey and white matter), and third, to investigate the SNR performance of DENSE when applied to the measurement of cardiac‐induced whole brain tissue motion. Specifically, the gain in SNR at 7 T compared with 3 T was determined, and from this a voxel‐wise uncertainty in the computed volumetric strain map was calculated.

## 2 MATERIALS AND METHODS

### 2.1 DENSE implementation

**Figure 1:**
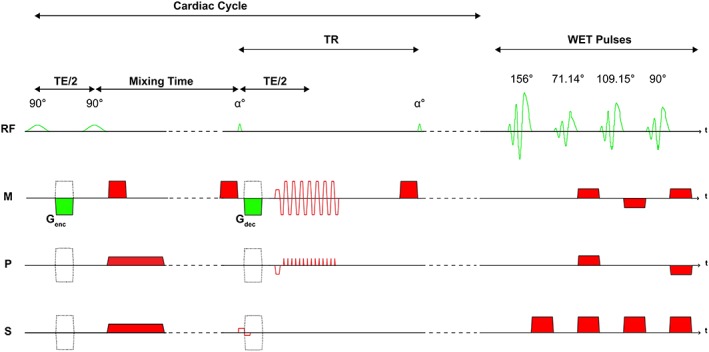
DENSE pulse sequence implemented in this study. The encoding gradients (*G*_enc_) and decoding gradients (*G*_dec_) were sequentially placed on each axis to encode motion along the RL, AP and FH directions, and were inverted in strength during acquisition of the second dynamic for phase subtraction (solid green areas and related dashed gradients). The spoiler gradients (red areas) performed during the mixing time on the axis hosting the encoding gradient were implemented with the same gradient moment as *G*_enc_ but with reversed strength, to avoid potential eddy current effects. *T*_1_‐ and *B*_1_‐insensitive suppression of any residual signal at the end of the cardiac cycle was achieved through the use of the WET RF pulses and dephasing gradients (red areas). This is meant to remove remaining tagged longitudinal magnetization prior to the next motion encoding block

A cardiac gated cine‐DENSE protocol comparable to that described by Soellinger et al^4^ was implemented in the MR scanner software to measure displacements in the right‐to‐left (RL), anterior‐to‐posterior (AP) and feet‐to‐head (FH) directions (see Figure 1 for the sequence schematic). In our study, the signal from fat tissue was suppressed through the use of a low bandwidth (500 Hz) for the two 90° RF pulses to avoid SENSE (sensitivity encoding) artefacts from high fat signal close to the receiver elements of the head coil. To suppress the effect of possible eddy currents, all spoiler gradients were inverted in strength relative to the motion encoding gradient. The residual longitudinal magnetization at the end of the last excitation in the cardiac cycle was suppressed through the use of water suppression enhanced through *T*_1_ effects (WET) pulses^17^ using the following four flip angles: 156°, 71.14°, 109.15°, and 90°. (This suppression was initially implemented to prevent potential higher order stimulated echoes from multiple successive motion encodings. However, it was later determined that the suppression is not necessary in practice for this application.)

The excitation flip angles were varied over time as described by Fischer et al.^18^ To calculate the flip angles, a *T*_1_ value of 1100 ms was used at 7 T, and 800 ms at 3 T. Preliminary results showed signal instability near the end of the cardiac cycle, so extra *T*_R_ values were included in the calculation to shift this instability outside of the desired range. We empirically determined that four extra *T*_R_ values were sufficient for this purpose, and consequently used flip angle sweeps with a maximum flip angle of 24.2°. The minimum angle (starting flip angle) depends on the number of cardiac phases, which differed between volunteers based on their heart rates. Table 1 contains further protocol parameters.

**Table 1:** Imaging parameters used in the study

Parameter	3 T DENSE	7 T DENSE	7 T *T*_1_‐weighted TFE
*D*_enc_ (mm) (FH/AP/RL)	0.175/0.0875/0.0875	0.175/0.0875/0.0875	—
Resolution (mm)	2.2 isotropic	2.2 isotropic	1.0 isotropic
FOV (mm) (FH × AP × RL)	250 × 250 × 190	250 × 250 × 190	300 × 250 × 190
*T*_R_ (ms) (FH/AP/RL)	36.8/38.3/38.3	36.4/37.3/37.3	4.2
*T*_E_ (ms)	10.2^a^	8.0	2.0
EPI phase encoding BW (Hz/pixel)	92.0	102.6	—
EPI factor	15	15	—
TFE factor	2	2	600
Readout BW (Hz/pixel)	2038.1	2796.0	505.1
Inversion delay (ms)	—	—	1280
Gradient strength (mT/m)	22.5	30	40
Gradient slew rate (T/m/s)	150	150	150
SENSE (AP × RL)	1.9 × 2	2 × 2.5	2 × 2.5
Flip angle (°)	Variable	variable^b^	7
*T*_1_ (ms)	800	1100	—
Triggering	ECG	ECG/POx (*n* = 1)^c^	—
Coverage of cardiac cycle (cardiac phases)	11–20	12‐20^c^	—
Scan duration (min)	3 × 5.5	3 × 4.5^d^	2

TFE, Turbo Field Echo; BW, Bandwidth.

a A slightly longer *T*_E_ was used at 3 T because of a different gradient performance in that MR system.

b The flip angles of the excitation RF pulse were increased over the cardiac cycle, using the field strength dependent *T*_1_.^18^

POx: a pulse oximeter unit was used because of artefacts in the VCG signal in one subject at 7 T.

c The number of cardiac phases depended on the heart rate, and covered approximately 110% of the cardiac cycle.

d The reported scan duration was for a heart rate of 60 bpm.

Pilot data on human volunteers were used to compare the DENSE implementation with the vendor‐provided phase contrast velocity mapping, as described in Reference 4, to validate the correctness of the motion encoding directions (including signs) and sensitivities for both the sequence implementation and processing software (see supplemental material for further details).

### 2.2 Measurements

#### 2.2.1 Tissue motion

The ethical review board (ERB) of the University Medical Center Utrecht approved the use of healthy volunteers for MRI protocol development. Eight healthy subjects (age 24 ± 5 years, five females) were included, after obtaining written informed consent, in accordance with the ERB approval. All subjects were scanned on both a 7 T and a 3 T scanner (Philips Healthcare, Best, The Netherlands) using a 32‐channel head coil at 7 T (Nova Medical, Wilmington, MA, USA) and a 16‐channel head coil at 3 T (Philips Healthcare), within a 2 h period per subject. Additionally, a slightly higher gradient strength of 30 mT/m was used at 7 T compared with 3 T (22.5 mT/m). Cardiac‐triggered DENSE was used to acquire whole brain volume displacement fields using a displacement sensitivity of 0.35 mm/*π* in the FH direction (direction with largest motion^4^) and 0.175 mm/*π* for the RL and AP directions. Seeking a parameter similar to *V*_enc_, which is used in phase‐contrast MRI, we designed *D*_enc_ to be the maximum displacement value in units of metres that produces a phase wrap in the final displacement map. Each encoding direction was acquired in two dynamics with opposing encoding gradients for phase error correction, yielding *D*_enc_ = 0.175 mm in the FH direction and *D*_enc_= 0.0875 mm for the other two directions.

Each DENSE acquisition at both 7 T and 3 T was triggered prospectively using a vectorcardiogram (VCG), which was performed every other heart cycle to allow magnetization recovery after the WET saturation pulses. A pulse oximeter (POx) was also connected as a backup triggering device in case the VCG triggering failed owing to magneto‐haemodynamic effects, which were more severe at 7 T.

The number of acquired cardiac phases was adjusted between subjects to cover 110% of the average heart cycle, depending on the individual heart rate. Arrhythmia rejection was used to minimize incorrect triggering from the VCG signal distortions and heart rate variability. The physiology traces from the VCG and POx devices were stored as text files by the scanner, and were saved after the examination for further analysis.

#### 2.2.2 SNR

For SNR analysis, noise images were acquired for the whole volume by repeating an entire right–left DENSE acquisition without RFs and gradients present. Additionally, a *T*_1_‐weighted 3D TFE scan was performed at 7 T to enable registration of the DENSE datasets and segmentation of white and grey matter tissues.

### 2.3 Analysis

#### 2.3.1 Tissue motion

The VCG and POx traces contained the trigger moments as recognized by the scanner software. In the case of severe magneto‐haemodynamic artefacts, triggering could be inconsistent, varying between triggers at the VCG R‐wave, and triggers at the slope of the artefact. All traces were visually analysed by comparing the trigger moments of the VCG relative to those of the POx. In the case of consistent triggering upon detection of the QRS complex, the VCG trigger was consistently before the POx trigger by about 0.2 to 0.3 s. Subjects with inconsistent triggering were excluded from the strain analysis.

Analysis of the acquired datasets was performed offline with custom MATLAB (MathWorks, Natick, MA, USA) software. Complex image pairs were generated from the phase images of the two dynamics with opposing gradients and the corresponding mean magnitude image. Through complex division, which yields phase subtraction, the motion‐sensitive phase information was obtained, with simultaneous cancellation of background phase errors (including any phase offsets induced from magnetic susceptibility changes, which may occur due to the magneto‐haemodynamic effect or due to fluctuations in the relative oxyhaemoglobin and deoxyhaemoglobin contents of the tissue). All phase images were unwrapped over time. Displacement was subsequently calculated from the unwrapped phase images using Equation 2 (see the appendix). Residual offsets in the displacement maps were corrected by subtracting the first acquired frame of the cardiac cycle from all other acquired frames.

All DENSE magnitude images for each subject were registered with a rigid transformation using elastix^19^ to the subject's respective *T*_1_‐weighted image space. The resulting transformation from the registration step was then applied to the corresponding displacement maps, yielding interpolated images with 1 mm isotropic voxel size.

A whole brain tissue mask free from skull or fat tissue was obtained for each subject from their respective *T*_1_‐weighted image as follows. First, the Computational Anatomy Toolbox (Jena University Hospital, Departments of Psychiatry and Neurology) for SPM12 (Wellcome Trust Centre for Neuroimaging, University College London) was used to segment the *T*_1_‐weighted image into CSF, grey matter and white matter probability maps. However, the DENSE images exhibited Echo Planar Imaging (EPI) distortions relative to the true anatomy reflected in the *T*_1_‐weighted images. Therefore, as a second step, the *T*_1_‐weighted images were registered non‐rigidly to the transformed DENSE magnitude images, and the resulting transformation from this registration step was then applied to the segmented probability masks. Finally, the grey and white matter 3D probability maps were summed to create a whole brain tissue mask, after which voxels in the summed tissue map with a probability less than 0.95 were removed from the mask.

Since a region with artefacts or CSF in any of the displacement maps could compromise the analysis of tissue volumetric strain, the 3D whole brain tissue mask was further modified to prevent these areas from being used in the analysis. Voxels with a non‐zero value in the CSF probability map were removed from the mask, thereby limiting potential partial volume effects. Artefacts were assumed to result in a non‐zero net displacement over the cardiac cycle. These areas were therefore estimated and removed from the whole brain tissue mask as follows. Two difference maps were generated for all encoding directions by subtracting displacements at the beginning and end of the cardiac cycle (*D*_net_) and by subtracting displacements from the first two acquired phases of the cardiac cycle (*D*_∆1_). For each encoding direction, the voxels in *D*_net_ whose absolute displacement exceeded three standard deviations of *D*_∆1_ were removed from the whole brain tissue mask. Subjects who had over 90% of their voxels removed due to the presence of artefacts were eliminated from the final analysis.

A 4D volumetric strain map was then created for each subject at each field strength by computing the divergence of their respective registered tissue displacement maps within the artefact‐free tissue mask. It should be noted that the divergence operation additionally eroded the masks, further ensuring that only tissue volumetric strain was evaluated. The mean of the volumetric strain map within the modified whole brain tissue mask was computed for each cardiac phase, allowing the creation of a volumetric strain curve for all subjects. As the POx trace is delayed with respect to the R‐wave peak of the VCG waveform, data from the subject triggered with the POx were shifted by 370 ms to correct for this delay during the normalization step. Each strain curve was resampled to 20 cardiac phases in the interval from 0 to 95% of the cardiac cycle of the respective subject.

To evaluate white and grey matter tissue volumetric strain differences, volumetric strain curves were obtained as described above but instead using grey and white matter masks separately. These were obtained by thresholding the respective probability maps at 0.95 and also modified to remove CSF and artefacts.

#### 2.3.2 SNR

4D SNR maps were created for each subject by dividing the magnitude image by the standard deviation of the noise, which was obtained by applying a moving standard deviation filter (15 × 15 × 15 pixels) to the real and imaginary noise images, and then taking the root sum of squares of the real and imaginary standard deviations.^20^ All SNR maps were transformed to each subject's respective *T*_1_‐weighted image space, in similarity with the displacement maps.

The SNR for each subject was evaluated by averaging the values contained in the registered SNR map, which was masked by the aforementioned whole brain tissue mask, which was not modified to remove artefacts (as artefacts were limited in the RL acquisitions), or CSF. The SNR of the first heart phase was used to determine the 7 T to 3 T SNR ratio. The SNR coefficient of variation (CoV) over the cardiac phases was calculated to assess the effectiveness of the variable flip angle approach. The mean SNR for all subjects was used to calculate the expected voxel‐wise uncertainty in the computed volumetric strain map (see Equation 7 in the appendix).

## 3 RESULTS

### 3.1 Tissue motion

**Figure 2:**
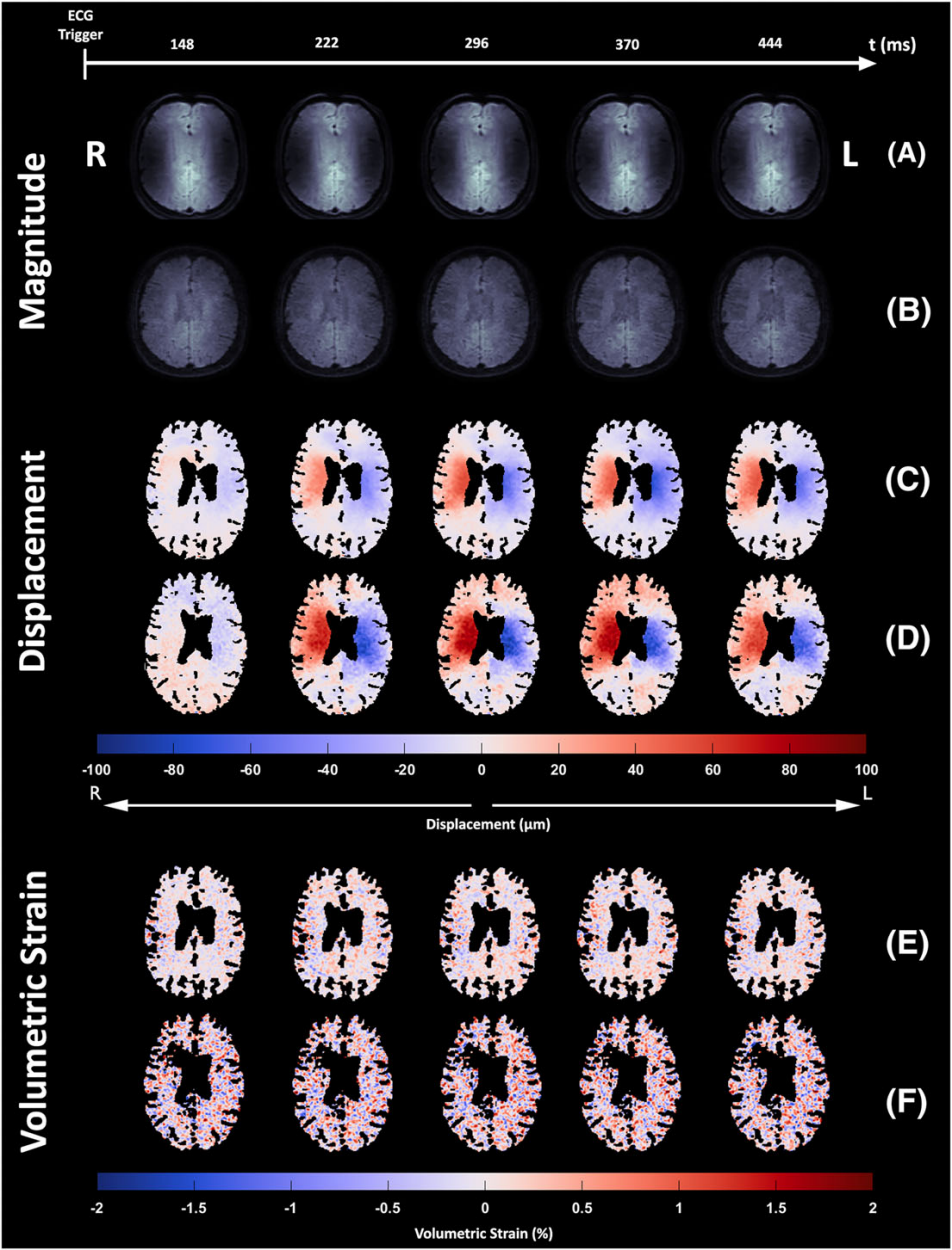
A‐D, DENSE RL magnitude (A, B) and displacement (C, D) images for the second to sixth acquired frames of the cardiac cycle of one subject at 7 T and 3 T, respectively. E, F, the corresponding volumetric strain maps for 7 T (E) and 3 T (F). While the displacement maps show the tissue mask free from CSF voxels, the volumetric strain maps shown here additionally reflect mask erosion due to the computation of spatial derivatives as well as the removal of noise/artefacts. The higher SNR at 7 T is apparent from the smoothness of the displacement maps. Anatomical left and right are indicated by L and R, hence the hemispheres are captured moving towards each other shortly after the ECG trigger

The DENSE acquisitions in all directions for all subjects were successfully accomplished at both 7 T and 3 T. However, analysis of the physiology data for two subjects at 7 T showed inconsistent VCG triggering between the three DENSE motion directions. Therefore, these two subjects were removed from the displacement and volumetric strain analyses at 7 T (no subjects at 3 T were found to have inconsistent VCG triggering). Additionally, one subject at 7 T and two subjects at 3 T were not included in the analysis because over 90% of their voxels (relative to the whole brain tissue mask) were removed by the artefact removal algorithm. Analysed subjects had mean 72.3 ± 5.3% (7 T) and 59.9 ± 16.6% (3 T) voxels remaining in the volumetric strain map. Thus, five subjects were analysed at 7 T and six at 3 T. Figure 2 shows example magnitude, displacement and volumetric strain images at both field strengths. The general observed brain tissue motion was towards the centre of the brain and down towards the spinal canal.

**Figure 3:**
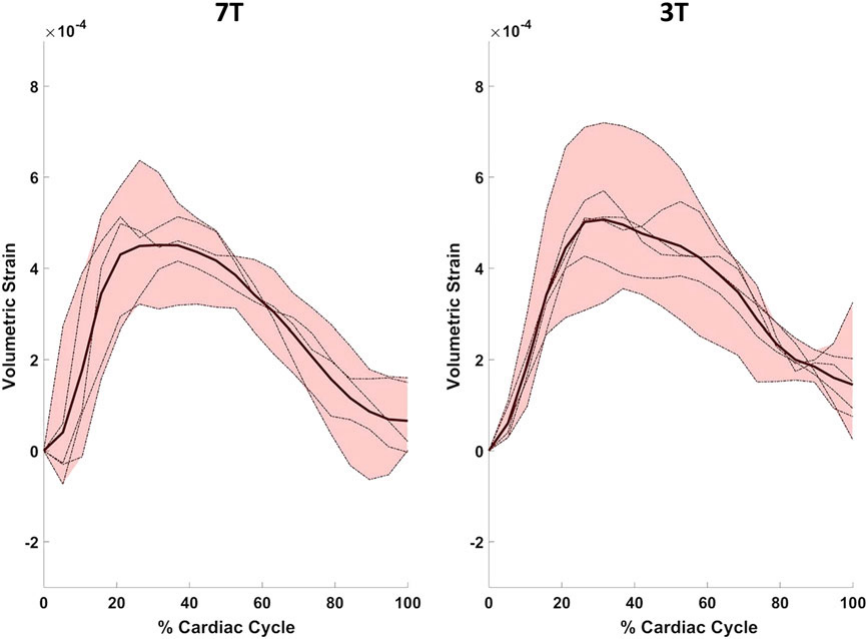
The mean whole brain tissue volumetric strain (bold lines) for all subjects over the cardiac cycle at 7 T and 3 T. the shaded region around the mean strain curve indicates the range between maximum and minimum variation among subjects. The dotted lines represent the individual strain curves for the subjects included in the strain analysis at either field strength (*n* = 5 at 7 T and *n* = 6 at 3 T)

**Figure 4:**
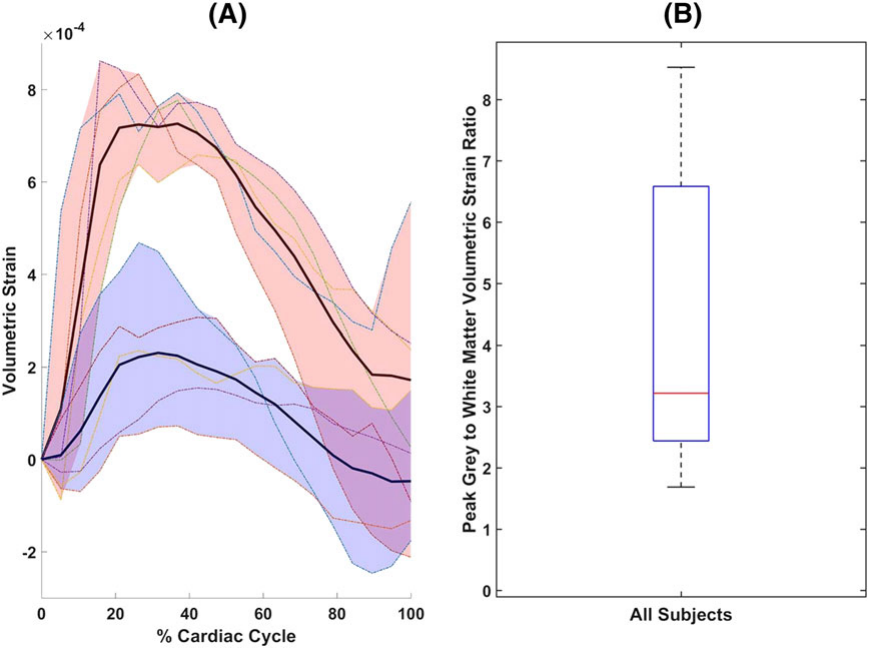
A, Grey and white matter mean volumetric strain curves (bold lines in red and blue shaded areas, respectively) for 7 T. the shaded region around the mean strain curves indicates the range between maximum and minimum variation among subjects for grey (red) and white (blue) matter. The coloured lines within the shaded regions represent the volumetric strain curve for the same individual subjects for the respective tissue types. B, box plot of the peak grey to white matter volumetric strain ratio, defined as the ratio of their respective values at the time of peak for the mean whole brain tissue curve (approximately 32% of the cardiac cycle). The whisker length of the box plot is set to display values exceeding ±2.7*σ* as outliers

The overall shape of the whole brain tissue volumetric strain curves was similar at both field strengths, with both attaining their peak value at approximately 32% of the cardiac cycle (Figure 3). Mean whole brain peak volumetric strains (mean ± SD) were (4.5 ± 1.0) × 10^−4^ and (5.1 ± 1.2) × 10^−4^ for 7 T and 3 T respectively. The peak grey and white matter volumetric strain was defined as their respective values at the time of peak for the whole brain tissue. At 7 T, the peak volumetric strain of grey matter was (7.2 ± 0.6) × 10^−4^, while for white matter the peak value was (2.3 ± 1.3) × 10^−4^ (Figure 4). The mean peak grey matter to white matter volumetric strain ratio was 4.4 ± 2.8 (median 3.2, inter‐quartile range, IQR, 4.1; see Figure 4B). A two‐sample *t*‐test for the difference in the means of the peak grey and white matter values (assuming unequal variances) was significant (*p* < 0.001). Similar values for peak grey and white matter volumetric strain were found at 3 T. At 3 T, the peak grey matter volumetric strain was (8.3 ± 2.4) × 10^−4^ and the peak white matter value was (2.1 ± 0.5) × 10^−4^, resulting in a mean peak grey matter to white matter volumetric strain ratio of 4.0 ± 1.2 (median 3.2, IQR 1.7). The difference between the means of grey and white matter peak volumetric strains (similarly assessed by a two‐sample *t*‐test) was also significant (*p* < 0.002).

### 3.2 SNR

**Figure 5:**
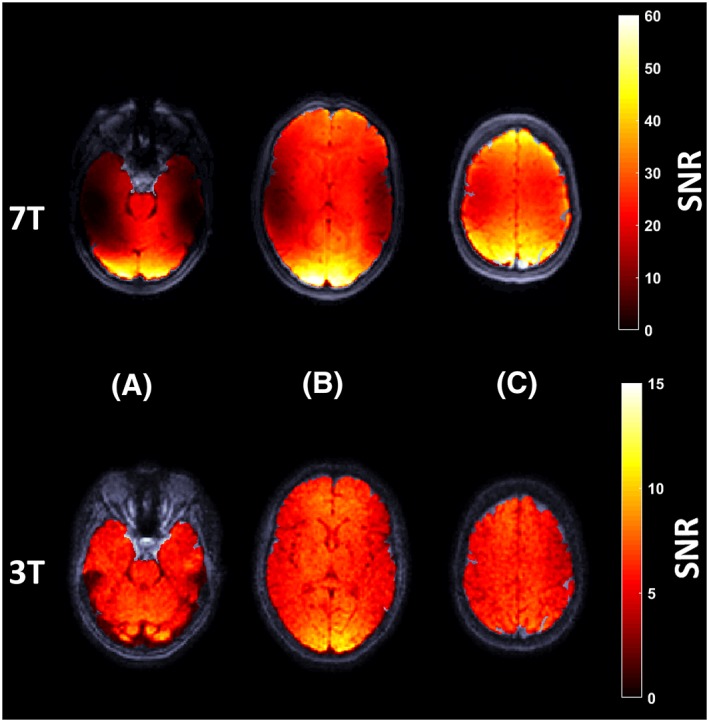
The masked SNR map overlaid on magnitude images for the same subject at 7 T and 3 T, here visualized in low (A), mid (B) and high (C) transverse slices of the brain. Note the larger colour range used here in the 7 T images due to the more than three times larger SNR present at 7 T. Greater SNR values were in general observed at the edge of the brain, corresponding to areas closer to the receive coils

**Figure 6:**
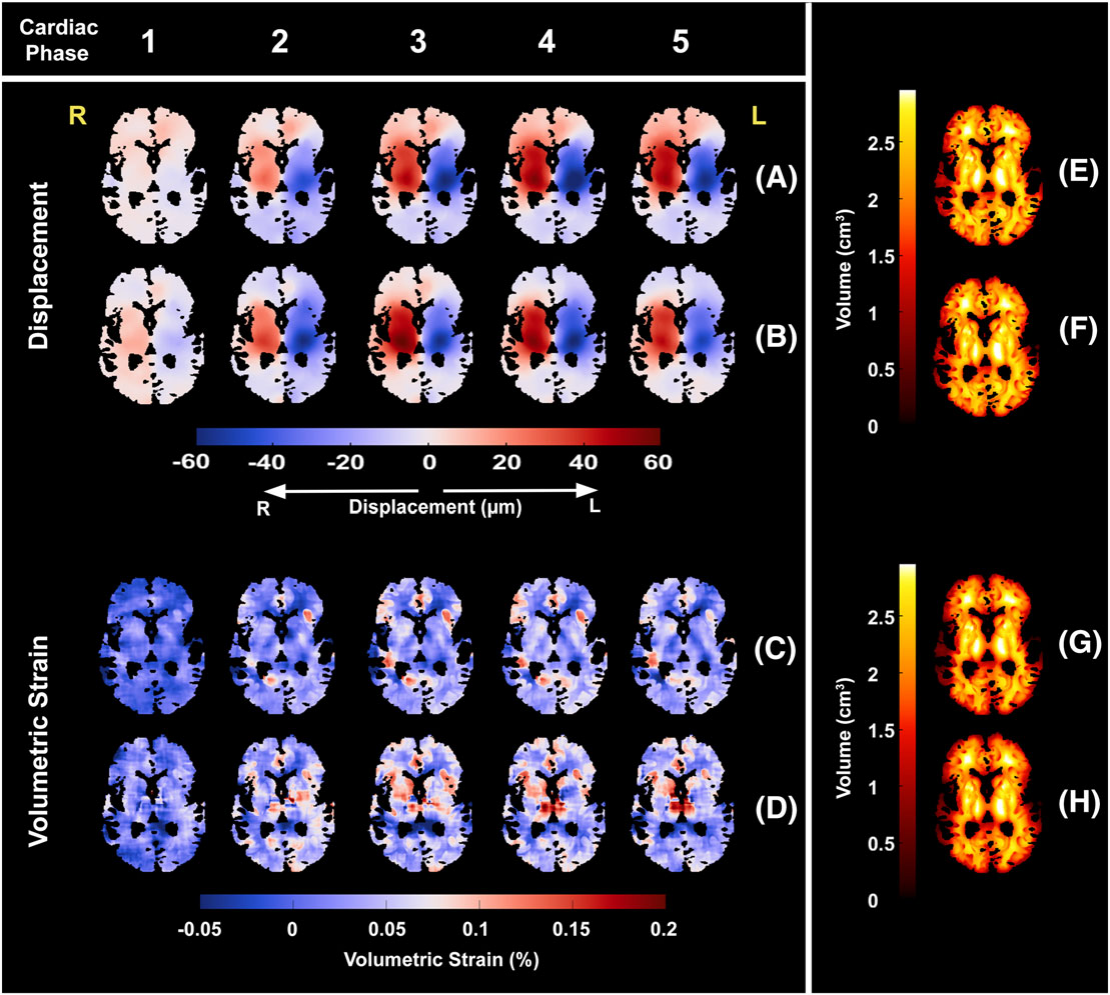
A‐D, example smoothed displacement and strain maps of the first five acquired cardiac phases for one subject at 7 T (A, C) and 3 T (B, D). smoothed displacement and strain maps were obtained by applying a 15 × 15 × 15 moving average filter to the separate grey and white matter masked maps (grey or white matter probability ≥0%, CSF 0%). The filtered grey and white matter maps were then combined into one map by weighting with their respective probability maps. Voxels in the strain map with absolute strains exceeding 1% were removed before smoothing. E‐H, the effective volume used within the moving average filter is shown for the displacement and strain maps at 7 T (E, G) and 3 T (F, H). The smoothed volumetric strain maps clearly show grey‐white matter contrast in various regions that is consistent between 3 T and 7 T measurements

The mean SNR for all subjects at 7 T and 3 T (mean ± SD) was 22.0 ± 7.3 and 7.0 ± 2.8 respectively. The mean 7 T to 3 T SNR ratio was 3.2 ± 0.7. Intensity changes over the cardiac cycle were limited, reflected by a low CoV, which was well below 10% for all subjects. The SNR at both field strengths was found to be higher at the edges of the brain closest to the receive coil (Figure 5). From Equation 7 (see appendix) the expected voxel‐wise uncertainty in the computed volumetric strain was found to be 1.4 × 10^−3^ and 4.4 × 10^−3^ for 7 T and 3 T, respectively. The uncertainty in the mean whole brain volumetric strain curves was smaller due to averaging over all subjects and over the number of voxels contained in the brain tissue masks, with values of 1.5 × 10^−6^ and 5.2 × 10^−6^ (mean ROI size 0.9 × 10^6^ and 0.7 × 10^6^ voxels) for 7 T and 3 T, respectively. Figure 6 shows the reduction in uncertainty of the displacement and volumetric strain maps when a 15 × 15 × 15 moving average filter is applied to the displacement and volumetric strain data within the grey and white matter masks separately.

## 4 DISCUSSION

### 4.1 Tissue motion

In this study, we implemented 3D cine‐DENSE at 7 T to assess the cardiac‐induced brain tissue volumetric strain in healthy subjects. We found a consistent pattern of tissue volume change amongst all subjects, with expansion (volume increase) occurring during the first 30% of the cardiac cycle. We also observed approximately four times higher peak volumetric strains of grey matter compared with white matter, which demonstrates the feasibility of DENSE as a tool to elucidate differences in the pulsatile behaviour of the brain's anatomical structures. Compared with 3 T, DENSE at 7 T yields three times higher SNR, which is currently not enough for a voxel‐wise analysis of the volumetric strain, as will be discussed further below.

The sharp, positive gradient of the strain curve between 0 and about 30% of the cardiac cycle suggests that volumetric strain reflects rapid expansion of the brain tissue due to a fast increase in blood volume of the small vessels within the tissue during systole, instead of tissue compression caused by the large vessels outside the brain tissue. The negative slope of the strain curve probably reflects relaxation of the brain tissue (due to outflow of venous blood) during diastole to its original state. The peak volumetric strain values found in this study show good agreement with other methods of evaluating brain volume changes over the cardiac cycle in healthy subjects. Hirsch et al^14^ found brain tissue peak volumetric strain values of (2.8 ± 1.9) × 10^−4^ for an ROI in white matter. Volumetric strain can equivalently be characterized in terms of the physical volume change if the initial volume is known. Applying the average volume used in the whole brain tissue mask as an estimate for the initial volume, the mean peak delta volume in this study was 0.47 mL ± 0.10 mL and 0.53 ± 0.13 mL for 7 T and 3 T respectively. Under the Monroe‐Kellie doctrine, these changes in brain tissue volume over the cardiac cycle should balance the CSF volume displaced into the spinal canal over the same time period.^6^, ^7^ The measured changes in brain tissue volume for this study compare favourably with the values of displaced CSF volumes reported in the literature (0.46 ± 0.15 mL, 0.51 ± 0.17 mL, 0.55 ± 0.12 mL, 0.58 ± 0.12 mL, 0.71 ± 0.32 mL, 0.77 ± 0.23 mL).^21^, ^22^, ^23^, ^24^, ^25^, ^26^ Changes in blood volume within the larger arteries over the cardiac cycle may also contribute to the caudally displaced CSF volume, making it difficult to disentangle the effects of arterial blood and brain tissue volume changes on spinal CSF volume dynamics. However, the expansion of the brain tissue appears to have a greater effect given the relative timings of CSF and blood outflow from the head in comparison with the termination of arterial expansion,^7^ thereby allowing reasonable comparison of cardiac‐induced brain tissue swelling with displaced spinal CSF volume. Additionally, compression of the venous system may also accommodate the expanding brain tissue and arterial volumes within the intracranial vault.

Volumetric strain is induced by the swelling of blood vessels within the incompressible tissue during systole,^7^, ^14^ and therefore also contains information on tissue stiffness. White matter has been observed to be stiffer than grey matter,^27^ which may explain the results of our study, where smaller volumetric strains were consistently found in the former in comparison with the latter. Thus, even under the assumption that the systolic blood volume change is proportional to the baseline blood volume, the ratio of the peak white and grey matter volumetric strains found in this study would not reflect the ratio of the blood volumes that occupies these two tissue types. Results found in the literature also support this view. Exploiting an MRI technique that combined arterial spin labelling and blood oxygen‐level dependent effects, Bulte et al obtained a grey‐to‐white matter blood volume ratio of 1.56.^28^ Vonken et al^29^ and Artzi et al^30^ utilized dynamic susceptibility contrast MRI to find a higher ratio of 1.87 and 2.38, respectively. The considerably higher ratio found in this study between these two tissue types thus demonstrates the entangled tissue blood volume change and stiffness information contained within the volumetric strain measurement.

The expansion of leptomeningeal arteries running within tightly folded sulci may also force apart the surrounding tissue. Such tissue displacements would still be incorporated within our tissue masks, and would result in an apparent volumetric strain that is not derived from the swelling of the small vessels. As CSF fills the sulci and fissures of the brain, the contribution of these areas containing ‘artificial tissue expansion’ to our estimations of brain tissue volumetric strain was therefore limited due to the removal of CSF regions from the analysis. However, the segmentation of CSF can be insufficient in regions where the grey matter is tightly folded, resulting in the retention of these areas within the volumetric strain map. The number of voxels where this occurs is difficult to quantify manually, but is expected to be small relative to the total number of voxels used in the estimation of small vessel pulsation. Therefore, while the contribution of these areas containing ‘artificial tissue expansion’ is probably suppressed through averaging, it nonetheless could also partially explain the larger volumetric strain observed in the grey matter compared with the white matter.

Owing to the difficulty of visualizing the small vessels in vivo, cSVD is typically assessed by imaging the lesions found within the brain parenchyma that arise from the disease. Volumetric strain is however a parameter map derived from the joint ‘mechanical’ contributions of both the viscoelastic tissue and the changing blood volume occupying the underlying small vessels. Thus, it may be well suited as a potential neuroimaging biomarker for cSVD, conceivably even before the onset of lacunar infarcts and white matter lesions, which characterize the disease, and also during its progression.^2^ In this case, these volumetric strain maps would offer a powerful tool for elucidating the pathophysiology of the disease. As the heart is the primary input to the studied intracranial dynamics, changes in cardiac function as a result of age or disease may also affect brain tissue volumetric strain measurements. Thus, normalization to cardiac function may be necessary to effectively evaluate any changes in brain tissue volumetric strain that would occur in the presence of cSVD. Additionally, the viscoelastic tissue properties can be derived from cardiac‐related displacement maps obtained by elastography.^1^ This would allow the changes in the volumetric strain due to the small vessels to be disentangled (at least partially) from the differences in tissue properties as assessed from elastography in cSVD.

Various cine‐MRI‐based approaches such as magnetic resonance elastography, phase contrast MRI and DENSE have been used to measure brain tissue pulsatility.^4^, ^7^, ^14^ Of note, the high availability of pre‐implemented phase contrast MRI sequences on the scanners of various MR vendors offers in principle a straightforward method to compute brain tissue displacement from the integrated velocity measurements and hence to calculate volumetric strain, provided that a very low velocity encoding (below 1 cm/s) is allowed by the user interface. However, strong bipolar gradients and short ramp times are necessary to encode the low velocities of brain tissue motion, which leads to relatively long repetition times due to gradient duty cycle limitations, and which can result in large phase errors due to eddy currents.^31^ Additionally, the integration procedure that is needed to compute the displacement fields from the velocity maps increases the uncertainty in the resulting strain map, potentially limiting its use in quantification of brain tissue expansion. Therefore, considering the much greater sensitivity to motion of DENSE compared with phase contrast MRI, DENSE appears to be a better overall tool for investigating the phenomenon of brain tissue expansion despite its inherently low SNR.

### 4.2 SNR

The acquisition of a separate noise map avoided typical problems in SNR calculations that can occur when the signal and noise values are from different spatial locations.^32^ By averaging over all voxels containing brain tissue, the SNR analysis yielded representative SNR values to study the field strength dependence. The observed SNR differences may be partially affected by the differing gradient performance and coils available on both MR systems. The spatial variation in SNR observed at 7 T may reflect the spatial inhomogeneity of the transmitted flip angles and the local sensitivity of the close‐fitting 32 receive coils. Nevertheless, at 7 T the SNR performance is probably improved over that at 3 T thanks to the greater number of coil elements^33^ and the closer fit of the receive coil to the subject's head. While this effect is minimized in the deeper areas of the brain, it may explain the 7 T to 3 T SNR ratio of 3.2 found in this study, which is higher than the expected value of 2.66. Although the higher SNR allows computation of a volumetric strain map with less uncertainty, the mean values for peak volumetric strain at 7 T and 3 T found in this study are approximately one order of magnitude lower than the calculated uncertainty per voxel, which currently limits the feasibility of a voxel‐wise analysis for the current resolution (2.2 mm isotropic).

A reduction of the noise in our DENSE measurements by about a factor of 10 would permit a voxel‐wise analysis of the peak volumetric strain map. This could be feasible (at the cost of temporal information) by acquiring a single cardiac phase at peak strain. In this case the use of a fixed 90° excitation pulse could be employed to vastly improve the SNR compared with the variable flip angle strategy used in this study.^34^ Further improvements in SNR through the use of specialized local surface coils may also facilitate a voxel‐wise analysis of peak volumetric strain in superficial brain regions. Also, de‐noising algorithms exist that show considerable improvement in other noisy MRI data such as cardiac motion^35^ and diffusion‐weighted imaging.^36^ These improvements remain the focus of future work.

Boosting the SNR by increasing the voxel size may also be a reasonable strategy for reducing the uncertainty in the volumetric strain map. Increasing the voxel size is more efficient than simply averaging over a greater number of smaller voxels. By way of illustration, averaging over two voxels can increase the SNR in the strain maps by $\sqrt{2},$ whereas increasing the voxel size by a factor of two to occupy a similar volume increases the SNR by a factor of $\;2\sqrt{2}$ (SNR_M_ increases by $\sqrt{2}$ and the derivative is more stable due to the larger distance between voxels, Equation 7 in appendix), while it even speeds up the acquisition by a factor of 2. However, this strategy of increasing the voxel size should be used with restraint to avoid partial volume effects. Finally, the use of other acquisition strategies, such as those employing a balanced displacement encoding^37^ may also boost the SNR efficiency.

The *B*_1_ inhomogeneity and *T*_1_ variation throughout the brain tissue limits the effectiveness of the variable flip angle approach as proposed by Fischer et al^18^ at both field strengths. Nonetheless, the low CoV values for all subjects indicates that the variable flip angle approach used in this study produces good signal stability over the cardiac cycle.

In this study we employed an EPI readout, as it has a high speed and high SNR efficiency,^38^ which is needed for this application. Consequently, we observed typical geometric EPI distortions (most visible in the prefrontal cortex) arising from off resonance spins due to the main magnetic field inhomogeneity. Using a spiral acquisition scheme may further increase the SNR,^39^ but currently requires offline reconstruction including compensation for the image blur from main field inhomogeneity, which may be considerable at 7 T. Unlike DENSE for cardiac applications, we did not observe typical EPI artefacts induced by fast motion, which also could be alleviated by spiral readouts.^40^ This is probably due to a generally lower amount of motion in the brain images, in combination with a very high motion encoding frequency, which effectively spoils the fast moving blood in the larger arteries.

Finally, a complete dataset of three orthogonal displacement measurements is required for volumetric strain computation. Image artefacts in any of these datasets, or de‐synchronicity between the three datasets, could cause inaccurate calculations. Similar to Soellinger et al,^4^ we observed artefacts in our DENSE images, which were most pronounced at the end of the cardiac cycle, and most severe in the FH displacement datasets. These artefacts were excluded from our volumetric strain. However, the whole brain tissue strain curve seems not to fully return to the initial value as one would expect for measurements of 100% of the cardiac cycle, which suggests imperfect artefact removal. Retrospective gating and/or motion navigators may reduce these artefacts while speeding up the acquisition. Further work is also needed to establish the reproducibility of this method and to develop a more time‐efficient, artefact‐free cine‐DENSE to evaluate the potential for investigating differences between healthy subjects and the ageing population or patients with cSVD. An interesting candidate for improving cine‐DENSE could come from combining compressed sensing and parallel imaging, a technique which was recently demonstrated for cardiac imaging.^41^

## 5 CONCLUSION

This study has demonstrated that DENSE at 7 T and 3 T allows the quantification of cardiac‐induced whole brain tissue motion, despite the inherently low SNR of the DENSE acquisition. The method was shown at 7 T to be sufficiently sensitive to detect physiological differences in volumetric strain, such as between grey and white matter in healthy human subjects, reflecting the difference in pulsatile behaviour between these two tissue types. Thus, the method holds potential for studying the blood volume pulsations of the cerebral small vessels, both under healthy conditions and in diseases such as cSVD.

## Supporting information

Data S1. Supporting information

## Appendix

The relationship between SNR and volumetric strain uncertainty (noise) is better understood through a voxel‐wise error propagation analysis. The volumetric strain is defined here as the regional change in tissue volume Δ*V* (due to a change in blood volume) relative to the original volume *V*: $\;\varepsilon_{v}=\frac{\Delta V}{V}$. Under the assumption of very small normal deformations, the dimensionless volumetric strain *ε*_*v*_ can be approximated from the divergence of the displacement field *D*:

(1) $$\varepsilon_{v}\approx \nabla \cdot D=\frac{\partial D_{x}}{\partial x}+\frac{\partial D_{y}}{\partial y}+\frac{\partial D_{z}}{\partial z}\;$$

where *∇* is the divergence operator and $\frac{\partial D_{x}}{\partial x},\frac{\partial D_{y}}{\partial y}\;\text{and}\;\frac{\partial D_{z}}{\partial z}\;$ are the partial derivatives of the displacement field *D* along orthogonal *x*, *y* and *z* directions, respectively. Each orthogonal component of *D* can be calculated from a pair of motion‐sensitive phase images acquired through DENSE by

(2) $$D_{x,y,z}=\frac{D_{{\mathrm{enc}}_{x,y,z}}}{\pi }\;{∆\varphi }_{x,y,z}\;$$

where *∆φ* is the difference image formed by the subtraction of the two displacement‐encoded phase images with opposite polarities of the encoding gradient to remove the background phase, and *D*_enc_ is the displacement encoding value in units of metres (similar to the *V*_enc_ parameter used in phase‐contrast MRI, which has units of metres/second).

Of interest is $\sigma_{\varepsilon_{v}}$, the resulting noise present in the volumetric strain map. The noise in a single displacement encoded phase image (which is motion encoded in one direction, say *x*), is related to the SNR in the corresponding magnitude image by

(3) $$\sigma_{\varphi }=\frac{1}{{\mathrm{SNR}}_{M}}$$

Assuming that the SNR_M_ values in the pair of phase images are the same, the subtraction step to remove the background phase increases the uncertainty (relative to a single phase image) by a factor of $\sqrt{2}$, i.e.

(4) $$\sigma_{∆\varphi }=\sqrt{2}\times \sigma_{\varphi }$$

However, because the phase images have opposite signs, subtraction yields signal accumulation and the SNR in the resultant image is increased by $\sqrt{2}$.

Given ∆*x*, the voxel size along *x*, we can define the strain in that direction as

(5) $$\varepsilon_{\mathrm{xx}\;\left(n\right)}≝\frac{D_{x}\left(n+1\right)-D_{x}\left(n-1\right)}{2∆x}$$

From Equations 2 and 5 the noise $\sigma_{\varepsilon_{\mathrm{xx}}}$ is thus

(6) $$\sigma_{\varepsilon_{\mathrm{xx}}}=\frac{\sqrt{2}\;}{2\;∆x}\frac{D_{{\mathrm{enc}}_{x}}\;}{\pi }\sigma_{∆\varphi }$$

A similar derivation can be made for the other two directions. Therefore, using Equations 3, 4 and 6, we find for the noise in the volumetric strain

(7) $$\sigma_{\varepsilon_{v}}=\frac{1}{\pi \;{\mathrm{SNR}}_{M}}\sqrt{\sum_{i=x,y,x}{\left(\frac{D_{{\mathrm{enc}}_{i}}\;}{∆i}\right)}^{2}}$$

Thus, an improvement in SNR_M_ reduces the presence of noise in the volumetric strain maps linearly. Similarly, improving the motion sensitivity *D*_enc_ in all directions linearly reduces $\sigma_{\varepsilon_{v}}$. However, increasing the voxel size has a more than linear effect due to the accompanying increase in SNR_M_.
