# Supplementary material for: Quantifying cardiac‐induced brain tissue expansion using DENSE
Source: NMR Biomed. 2018 Dec 21;32(2):e4050. doi: 10.1002/nbm.4050 (PMC6519010; doi:10.1002/nbm.4050)
Supplement: Supplementary file 1 — Data S1. Supporting information [file NBM-32-e4050-s001.DOCX]

**Supplementary Materials**

Comparison of tissue motion measured by DENSE to measurements by PC-MRI

**Introduction**

The investigation of cardiac-induced brain tissue volumetric strain as proposed in this study requires the acquisition of three orthogonally oriented displacement maps of brain tissue motion over the cardiac cycle. Naturally, the correct interpretation of the tissue volumetric strain (i.e. the amount of tissue expansion or compression) depends upon the correctness of the amplitudes and signs of the displacement maps used to compute the volumetric strain. To assess the correctness of the amplitudes and signs of brain tissue displacements obtained from our implementation of displacement encoding using stimulated echoes (DENSE), we compared velocity maps derived from these measurements with a vendor implemented phase contrast MRI (PC-MRI) acquisition.

**Method**

Velocity maps were acquired in the same directions as the DENSE measurements (Right-Left (RL), Anterior-Posterior (AP) and Feet-Head (FH)) using a vendor implemented PC-MRI acquisition. See table 1 for PC-MRI scan parameters, which were designed to be comparable to those used in the DENSE acquisition. The two acquisitions were performed in the same subjects and data was acquired during the same scan session.

We then derived velocity maps from three DENSE measurements by taking the temporal derivative of the motion maps, and compared the DENSE derived velocity maps to the PC-MRI based velocity maps after temporal alignment. The PC-MRI measurements were acquired retrospectively, using the signal from a pulse oximeter placed on the finger to synchronize the acquisitions to the cardiac cycle. The signal from the pulse oximeter is delayed relative to the VCG signal used to trigger the DENSE acquisitions. This is due to the delay between the ventricular depolarisation at the heart (which gives a characteristic signal peak used in VCG prospective triggering) and the detection of the arterial pulse wave as it arrives at the pulse oximeter located at the finger. Therefore, after interpolating the DENSE-derived and PC-MRI mean velocity curves to the same temporal grid, the PC-MRI was shifted by approximately 215ms to account for this delay, thereby better aligning the time to peak of both curves.

Our analysis consisted of assessing, first, the general agreement of the global spatial patterns of the tissue velocity between the two acquisition methods, and second, the agreement of the mean velocity measured over the cardiac cycle within a region of interest (ROI) (see Figure 1).


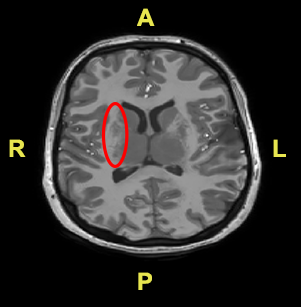


**Figure 1.** High resolution T1 image showing the approximate location of the ROI used to obtain the mean velocity curves. The ROI was placed at a location that best illustrates the unique profiles of the mean velocity for each measured direction.


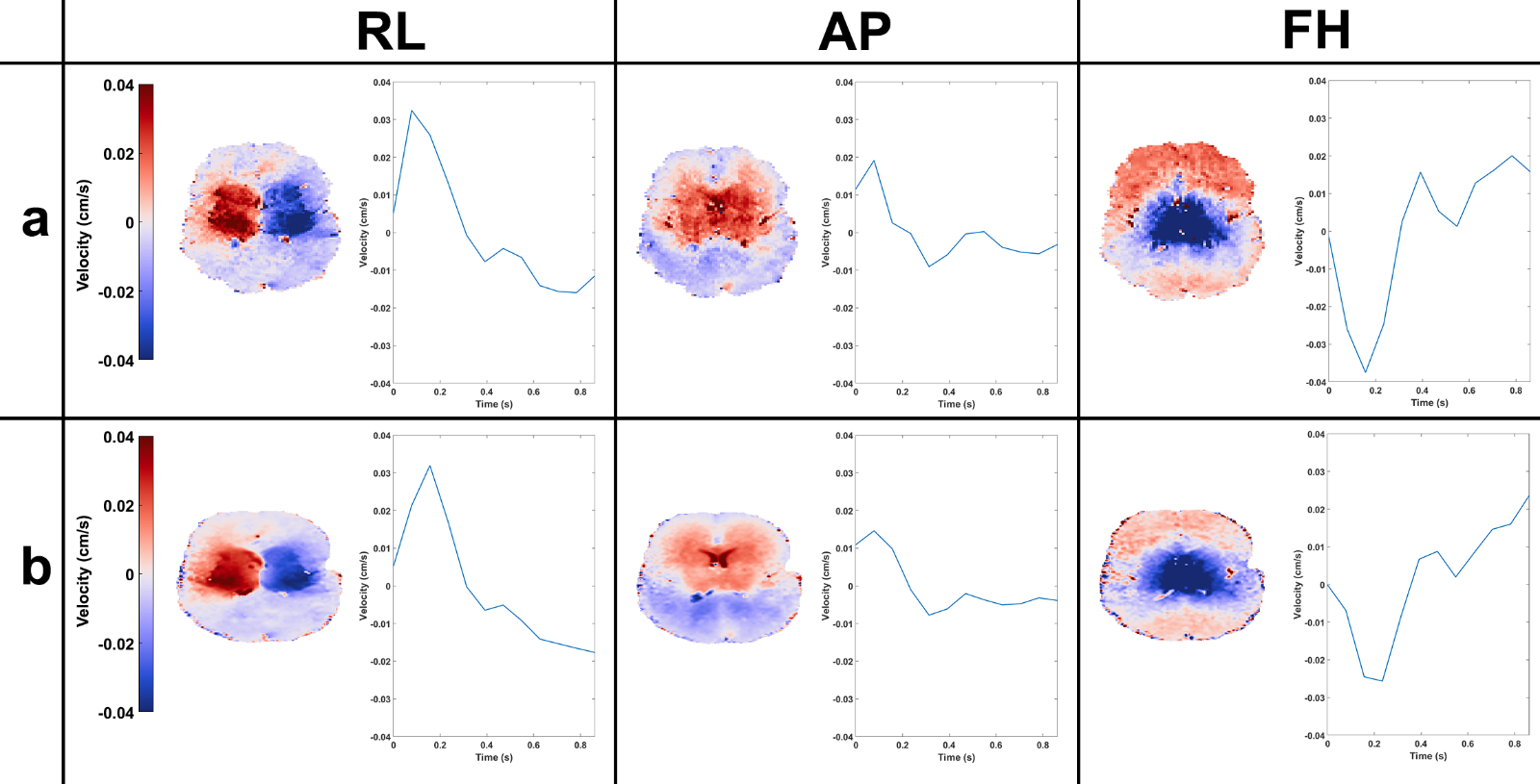


**Figure 2.** a) RL, AP and FH PC-MRI brain tissue velocity maps of a subject shown at the moment of peak velocity measured over the cardiac cycle for each respective direction. The corresponding mean velocity curve for the ROI indicated in Figure 1 is also shown, for all acquired phases of the cardiac cycle. b) DENSE-derived velocity maps of the same subject in the RL, AP and FH directions, also shown at the moment of peak velocity. The corresponding mean velocity curve for the ROI indicated in Figure 1 is also shown. The PC-MRI images are shown with the acquired resolution (2.3 mm isotropic), while the DENSE-derived velocity maps shown here were registered and interpolated (without smoothing) to the subject’s high resolution T1 image (1.0 mm isotropic). This may partially explain the smoother appearance of the DENSE velocity maps in comparison to the PC-MRI images, although most of the smooth appearance is due to the high SNR of the DENSE velocity maps. Positive (red) motion is directed towards the Left, Posterior and Head directions, respectively.

**Results & Discussion**

The general spatial patterns of DENSE-derived and PC-MRI velocity maps were found to agree for all directions measured, see Figure 2. This confirms that the implementation of the DENSE technique was correct. The general shape and amplitudes of the mean-velocity curves at the selected ROI for both methods were also in agreement.

The primary peak (or dip for the FH dataset due to the negative velocities) observed in the mean velocity curves for all directions is likely related to tissue motion induced by the arterial pulse wave during systole^1^, while the secondary peak (or dip in FH) possibly arises from the energy deposited to the brain by the reflected arterial pulse wave.

At the moment of peak velocity, the brain tissues were observed moving together towards the midline and down towards the spinal canal. This is consistent with the patterns of cardiac-induced tissue motion reported in the literature^1–4^. It is unclear as to why Soellinger et al. reported motion of the cerebral hemispheres away from each other in the right-left direction, despite also comparing their VCG triggered DENSE measurements to PC-MRI^5^.

**Conclusion**

We found good agreement between PC-MRI and DENSE-derived velocity maps of brain tissue motion. The agreement between the two methods supports the notion that the DENSE displacement measurements used in this study had the correct signs and amplitudes. This strengthens the finding of mean positive whole brain tissue volumetric strain over the cardiac cycle (reflecting expansion of brain tissue due to swelling of the microvasculature) and the associated amount of peak volume change reported in this study.

**References**

1. Greitz D, Wirestam R, Franck A, Nordell B, Thomsen C, Stahlberg F. Pulsatile brain movement and associated hydrodynamics studied by magnetic resonance phase imaging. *Neuroradiology*. 1992;34(5):370-380. doi:10.1007/BF00596493

2. Poncelet BP, Wedeen VJ, Weisskoff RM, Cohen MS. Brain parenchyma motion: measurement with cine echo-planar MR imaging. *Radiology*. 1992;185(3):645-651. doi:10.1148/radiology.185.3.1438740

3. Weaver JB, Pattison AJ, McGarry MD, et al. Brain mechanical property measurement using MRE with intrinsic activation. *Phys Med Biol*. 2012;57(22):7275-7287. doi:10.1088/0031-9155/57/22/7275

4. Hirsch S, Klatt D, Freimann F, Scheel M, Braun J, Sack I. In vivo measurement of volumetric strain in the human brain induced by arterial pulsation and harmonic waves. *Magn Reson Med*. 2013;70(3):671-683. doi:10.1002/mrm.24499

5. Soellinger M, Rutz AK, Kozerke S, Boesiger P. 3D cine displacement-encoded MRI of pulsatile brain motion. *Magn Reson Med*. 2009;61(1):153-162. doi:10.1002/mrm.21802

**Table 1.** PC-MRI imaging parameters used to evaluate the correctness of DENSE displacement signs and amplitudes.

| **Parameter** | **Value** |
| --- | --- |
| V_enc_ (mm/s) | 3 |
| Resolution (mm) | 2.2 isotropic |
| FOV (mm) (FHxAPxRL) | 250 x 250 x 190 |
| TR (ms) | 40 |
| TE (ms) | 25 |
| EPI factor | 15 |
| Readout BW (Hz/pixel) | 2258.4 |
| EPI Phase Encoding BW (Hz/pixel) | 84.2 |
| Max. gradient strength (mT/m) | 24 |
| Max. gradient slew rate (T/m/s) | 100 |
| SENSE (AP x RL) | 2.7 x 2.5 |
| Flip angle (deg) | 15 |
| Triggering | Retrospective, (pulse oximeter) |
| Scan duration (at 60 beats per minute) | 3 x 2.3 mins |
